# Glyceollins trigger anti-proliferative effects through estradiol-dependent and independent pathways in breast cancer cells

**DOI:** 10.1186/s12964-017-0182-1

**Published:** 2017-06-30

**Authors:** Sylvain Lecomte, Frederic Chalmel, François Ferriere, Frederic Percevault, Nicolas Plu, Christian Saligaut, Claire Surel, Marie Lelong, Theo Efstathiou, Farzad Pakdel

**Affiliations:** 1 Institut de Recherche en Santé-Environnement-Travail (IRSET), University of Rennes 1, 9 Avenue du Pr Léon Bernard, 35000 Rennes, France; 2 Inserm U1085, Team Transcription, Environment and Cancer, 9 Avenue du Pr Léon Bernard, 35000 Rennes, France; 3 Inserm U1085, Team Viral and Chemical Environment & Reproduction, 9 Avenue du Pr Léon Bernard, 35000 Rennes, France; 4 Laboratoire Nutrinov, Technopole Atalante Champeaux, 8 rue Jules Maillard de la Gournerie, 35012 Rennes Cedex, France

**Keywords:** Glyceollins, Estrogen receptors, Cell proliferation, Transcriptomic, Gene expression, Breast cancer

## Abstract

**Background:**

Estrogen receptors (ER) α and β are found in both women and men in many tissues, where they have different functions, including having roles in cell proliferation and differentiation of the reproductive tract. In addition to estradiol (E2), a natural hormone, numerous compounds are able to bind ERs and modulate their activities. Among these compounds, phytoestrogens such as isoflavones, which are found in plants, are promising therapeutics for several pathologies. Glyceollins are second metabolites of isoflavones that are mainly produced in soybean in response to an elicitor. They have potentially therapeutic actions in breast cancer by reducing the proliferation of cancer cells. However, the molecular mechanisms driving these effects remain elusive.

**Methods:**

First, to determine the proliferative or anti-proliferative effects of glyceollins, in vivo and in vitro approaches were used. The length of epithelial duct in mammary gland as well as uterotrophy after treatment by E2 and glyceollins and their effect on proliferation of different breast cell line were assessed. Secondly, the ability of glyceollin to activate ER was assessed by luciferase assay. Finally, to unravel molecular mechanisms involved by glyceollins, transcriptomic analysis was performed on MCF-7 breast cancer cells.

**Results:**

In this study, we show that synthetic versions of glyceollin I and II exert anti-proliferative effects in vivo in mouse mammary glands and in vitro in different ER-positive and ER-negative breast cell lines. Using transcriptomic analysis, we produce for the first time an integrated view of gene regulation in response to glyceollins and reveal that these phytochemicals act through at least two major pathways. One pathway involving FOXM1 and ERα is directly linked to proliferation. The other involves the HIF family and reveals that stress is a potential factor in the anti-proliferative effects of glyceollins due to its role in increasing the expression of REDD1, an mTORC1 inhibitor.

**Conclusion:**

Overall, our study clearly shows that glyceollins exert anti-proliferative effects by reducing the expression of genes encoding cell cycle and mitosis-associated factors and biomarkers overexpressed in cancers and by increasing the expression of growth arrest-related genes. These results reinforce the therapeutic potential of glyceollins for breast cancer.

**Electronic supplementary material:**

The online version of this article (doi:10.1186/s12964-017-0182-1) contains supplementary material, which is available to authorized users.

## Background

Breast cancer is the most prevalent cancer in women worldwide and has an incidence of 89.7 per 100,000 women in Western Europe. The WHO estimates that breast cancer was responsible for over 500,000 deaths in 2011 [1]. Among the different types of breast cancer, the most common is estrogen receptor (ER)-positive cancer, which represents approximately 80% of diagnosed cases of cancer. The ER belongs to the nuclear receptor superfamily and is divided into two subtypes, ERα and ERβ. The ER acts in cells by directly binding to DNA on a responsive element called the estrogen-responsive element (ERE) or by interacting with other transcription factors, such as stimulating protein 1 (Sp1) or activator protein 1 (AP1), which are already bound to responsive elements in promoter regions. The ER also modulates signaling pathways such as the MAPK and PI3K/AKT pathways [2]. Thus, ERα, the major isoform in breast tissue, plays an essential role in normal mammary gland development and function as well as in breast cancer initiation and growth. ERs are bound by the natural hormone estradiol (E2), which has a pleiotropic effect and is responsible for the proliferation and survival of breast epithelial cells. Therefore, E2 plays an important role in breast cancer growth. However, E2 is also essential for maintaining cell differentiation, which consequently limits metastatic potential. Hence, ERα is a good prognostic marker and a prime target for therapy. Endocrine therapies such as tamoxifen or fulvestrant are effective for ER-positive cancer, but frequent relapses are observed [3]. Currently, the focus is on the discovery of new compounds with selective ER-modulator (SERM) activities. Phytoestrogens appear to be promising candidates and have been well studied [4]. Phytoestrogens could be ERα agonist or antagonist depending of cellular type and phenotype studied such as cell proliferation and differentiation [5]. Among this family, pterocarpans, which are second metabolites of isoflavones and the best-known members are glyceollins, have been studied since the 2000s.

Glyceollins are phytoalexins produced mainly by soybeans after elicitation by different types of stressors, such as UV, low temperatures or microorganisms. The glyceollin family includes three compounds: glyceollin I (GI), glyceollin II (GII) and glyceollin III (GIII). In plants, glyceollins are involved in host defense against pathogens such as fungi [6, 7] or nematodes [8]. Glyceollins are promising therapeutic compounds for numerous human pathologies, including breast cancer [9]. Interactions between glyceollins and ERα or ERβ were described for the first time in 2000 [10]. Glyceollins act as antiestrogenic compounds that directly interact with both ER isoforms [11, 12], and they have the capacity to suppress tumorigenesis of breast and ovarian cancer [13]. Glyceollin I is the most potent antiestrogenic molecule, and docking experiments have shown that glyceollin I can interact with the ER in a similar manner to tamoxifen to exert antagonist activity [12]. Thus, chemically synthesized glyceollin I was generated and assessed. It was shown that the natural enantiomer exerted anti-proliferative activities against numerous cell lines, including ER-positive breast cancer cells [14], and that the compound is a potent inhibitor of ER activation [15].

However, the precise antiestrogenic mechanisms associated with glyceollins in ER-positive breast cancer remain elusive. In this work, we synthetized natural enantiomers of glyceollin I and II to determine their impact in vivo on the growth of galactophore ducts in mouse mammary glands as well as in vitro in different breast cell lines. The ability of glyceollins to bind and activate ERs as well as their effects on the expression of endogenous E2-dependent genes were assessed. Glyceollins showed surprising effects on gene expression, which led us to perform transcriptomic analysis of the ER-positive breast cell line MCF-7 to better elucidate the mechanisms underlying the actions of these compounds. We found that glyceollins exert their effects through both ER-dependent and ER-independent pathways involving different transcription factors.

## Methods

### Animals

Ovariectomized (ovx) (at 4.5 weeks) and intact non-ovariectomized (non-ovx) RjOrl SWISS female mice were purchased from Janvier Labs. The animals were housed at 20 ± 2 °C under 60 ± 10% humidity with a 12 h light/dark cycle. After 3 weeks of acclimation, 9-week-old mice were weighed and randomly assigned to one of 11 groups (Table 1) of at least six mice each with similar average body weights. Treatment (Table 1) was delivered by subcutaneous injection (sc) at 24 h intervals for 3 consecutive days with sesame oil as a vehicle. On day 3, the animals were weighed and then euthanized by cervical dislocation. Uteri and mammary glands were removed.

**Table 1:** Description of randomized mouse groups and their treatments

Mice groups	Treatment
Non-Ovariectomized (non-Ovx)	Vehicle
Ovariectomized (Ovx)	Vehicle
Estradiol (E2)	E2 10 μg/kg
Glyceollin I Low (GI L)	GI 50 mg/kg
Glyceollin II Low (GII L)	GII 50 mg/kg
Estradiol + Glyceollin I Low (E2 + GI L)	E2 10 μg/kg + GI 50 mg/kg
Estradiol + Glyceollin II Low (E2 + GII L)	E2 10 μg/kg + GII 50 mg/kg
Glyceollin I High (GI H)	GI 100 mg/kg
Glyceollin II High (GI H)	GII 100 mg/kg
Estradiol + Glyceollin I High (E2 + GI H)	E2 10 μg/kg + GI 100 mg/kg
Estradiol + Glyceollin II High (E2 + GII H)	E2 10 μg/kg + GII 100 mg/kg

### Uterotrophic measurements

The uterus was carefully dissected at the level of the vaginal fornix, trimmed of fascia and fat, gently blotted on moistened filter paper and weighed.

### Mammary whole-mount preparations and immunostaining

The first inguinal mammary fat pads were removed and stained as described by Tian et al. [16]. Briefly, mammary fat pads were spread as flat as possible on a glass surface and fixed with 4% paraformaldehyde. For assessment of epithelial duct length, the mammary glands were stained overnight in carmine alum (0.2% carmine, 0.5% aluminum potassium sulfate), dehydrated in an ethanol gradient and clarified overnight in xylene. Tissues were then photographed under a SteREO Discovery V8 microscope (Zeiss, original magnification, ×1). For Ki-67 and Epcam immunostaining, mammary fat pads were embedded in Tissue Tek mounting medium (Sakura) and sliced with a cryostat. The slices were then incubated at room temperature with a rabbit anti-Ki-67 antibody (Abcam) and a rat anti-Epcam (sc53532, Santa Cruz) for 1 h in PBS supplemented with 0.3% Triton ×100 and 0.5% milk. A dye-conjugated secondary antibody was then incubated with the sections at room temperature for 1 h in PBS supplemented with 0.3% Triton ×100 and 0.5% milk. Images were obtained with an Imager.Z1 ApoTome AxioCam (Zeiss) microscope and processed with Axio Vision Software. The percentage of Ki-67-positive cells was determined by counting the total numbers of ductal epithelial cells and Ki-67-positive cells using ImageJ software.

### Cell culture and reagents

MCF-7 cells were maintained in DMEM, 4.5 g/L glucose supplemented with non-essential amino acids (NEAA) (Invitrogen) and 10% fetal bovine serum (FBS) (Biowest). T47D cells were maintained in RPMI 1640 supplemented with NEAA, sodium pyruvate (Invitrogen) and 10% FBS (Biowest). HC-11 cells were maintained in RPMI 1640 supplemented with 2 mM L-glutamine, 5 μg/mL insulin (Invitrogen), 0.01 μg/mL epidermal growth factor (EGF) (Abcys) and 10% FBS (Biowest). MCF10-A cells were maintained in DMEM/F12 supplemented with 0.5 μg/mL hydrocortisone, 10 μg/mL insulin (Invitrogen), 20 ng/mL EGF (Abcys), 100 ng/mL cholera toxin (Sigma) and 5% horse serum (Invitrogen) All cell lines were cultured with penicillin/streptomycin (Invitrogen) at 37 °C under 5% CO_2_. For steroid treatments, cells were cultured for at least 24 h in steroids and serum-free DMEM without phenol red and with 2.5% or 5% charcoal/dextran-stripping FBS (Biowest) for MCF-7 and T47D cells, respectively. E2 was purchased from Sigma. Natural enantiomers (6aS and 11aS asymmetric carbon configurations) of glyceollin I and glyceollin II were chemically synthesized by HPC Pharma adapting the synthesis method described by Khupse et al. [17] and Luniwal et al. [18]. The purity was determined at 98% and 99% for glyceollin I and glyceollin II, respectively.

### Proliferation assay

Cells (7500 cells/well for HC-11, 20,000 cells/well for MCF-7 and MCF10-A, and 40,000 cells/well for T47D) were plated in 24-well plates and then deprived of steroids and serum for 72 h. The cells were treated with different doses of glyceollin I or II with or without 10^−9^ M E2 for 6 days with renewal of the treatment mixture on day 3. After treatment, the cells were trypsinized, and the cell number was determined using a TC10 Automated Cell Counter (Bio-Rad).

### Luciferase assay

MCF-7 cells (30,000 cells/well) were plated in 24-well plates. After serum and steroid deprivation, the cells were transfected overnight with 100 ng of an ERE-TK-luciferase vector, which encodes luciferase under the control of one ERE, and with 20 ng of a CMV-β galactosidase vector, which served as a control of transfection efficiency control. JetPEI was used as a transfection reagent (Polyplus transfection). Next, the cells were treated with 10^−9^ M E2 and/or with different doses of glyceollin I or II. ICI_182.780_ (Tocris) was used as ER-inhibitor. The cells were lysed in Passive Lysis Buffer (Promega), and luciferase activity was determined using a commercial luciferase assay system (Promega).

### RNA extraction and real-time PCR

MCF-7 cells (250,000 cells/well) were plated in 6-well plates. After 30 h of serum and steroid deprivation, the cells were treated with solvent as a control, with 10^−9^ M E2 or with different concentrations of glyceollin I or II. Total RNA was extracted using a RNeasy mini kit (Qiagen) according to the manufacturer’s instructions. Then, the RNA was reverse-transcribed using an M-MLV RT kit (Invitrogen) according to the manufacturer’s instructions. For real-time PCR, 5 ng of cDNA was used with 150 nM primers (Table 2) and iTaq Universal SYBR Green Supermix (BioRad). Real-time PCR was performed on a CFX 384 apparatus, and the results were analyzed with CFX Manager software (BioRad).

**Table 2:** Gene names and primer sequences used in real-time PCR experiments

Gene name and symbol	Forward primer	Reverse primer
Progesterone receptor (PgR)	CCCGCCGTCGTAACTTTGG	GTGCCTATCCTGCCTCTCAATC
Growth regulation in breast cancer 1 (GREB1)	GAGGATGTGGAGTGGAGACC	CAGTACCTCAAAGACCTCGGC
Trefoil Factor 1(TFFI/pS2)	ACCATGGAGAACAAGGTGA	CCGAGCTCTGGGACTAATCA
Amphiregulin (AREG)	GTATTTTCACTTTCCGTCTTGTTTTG	CCTGGCTATATTGTCGATTCA
Forkhead box M1 (FOXM1)	AGCGAGACCCATCAAAGTGG	GGTCTTGGGGTGGGAGATTG
Estrogen receptor 1 (ESR1/ERα)	TTTATGGGAAAAGGCTCAAA	GACAAAACCGAGTCACATCA
Estrogen receptor 2 (ESR2/ERβ)	AGAGTCCCTGGTGTGAAGCAAG	GACAGCGCAGAAGTGAGCATC
FBJ murine osteosarcoma viral oncogene homolog (FOS)	GAATTAACCTGGTGCTGGAT	GAACACACTATTGCCAGGAA
Peroxisome proliferator-activated receptor gamma (PPARG)	GCAATCAAAGTGGAGCCTGC	CCCTTGCATCCTTCACAAGC
Hypoxia inducible factor 1, alpha subunit (HIF1A)	CTGCCACCACTGATGAATTA	GTATGTGGGTAGGAGATGGA
Endothelial PAS domain protein 1 (EPAS1/HIF2α)	GCGCTAGACTCCGAGAACAT	TGGCCACTTACTACCTGACCCTT
Vascular endothelial growth factor A (VEGFA)	AGGAGGAGGGCAGAATCATCA	CTCGATTGGATGGCAGTAGCT
DNA-damage-inducible transcript 4 (DDIT4/REDD1)	AGGAAGCTCATTGAGTTGTG	GGTACATGCTACACACACAT
Nuclear Receptor Subfamily 2 Group F member 1 (NR2F1/COUP-TFI)	TACGTGAGGAGCCAGTACCC	CGATGGGGGTTTTACCTACC
Chemokine (C-X-C motif) receptor 4 (CXCR4)	GCCTTATCCTGCCTGGTATTGTC	GCGAAGAAAGCCAGGATGAGGA
Atypical chemokine receptor 3 (ACKR3/CXCR7)	ACAGGCTATGACACGCACTG	ACGAGACTGACCACCCAGAC
Chemokine (C-X-C motif) ligand 12 (CXCL12)	CTCCTGGGGATGTGTAATGG	GCCTCCATGGCATACATAGG
Glyceraldehyde-3-phosphate dehydrogenase (GAPDH)	GGGCATCCTGGGCTACACTG	GGGCATCCTGGGCTACACTG
TATA box binding protein (TBP)	TGCACAGGAGCCAAGAGTGAA	CACATCACAGCTCCCCACCA

### Transcriptomic analysis

MCF-7 cells (250,000 cells/well) were plated in 6-well plates. After 30 h of serum and steroid deprivation, the cells were treated for 24 h with solvent as a control, with 10^−9^ M E2, with 10^−5^ M glyceollin I or II, or with an E2 and glyceollin I or II co-treatment. Total RNA was prepared as described above. RNA quantity and purity were determined using a Nanodrop (Thermo Fisher). Only RNA samples with 260/280 and 260/230 ratios >1.8 were selected. RNA quality was analyzed with a bioanalyzer (Agilent), and RNA samples with a RIN > 8.5 and an 18S/28S ratio > 1.7 were selected for spotting on a SurePrint G3 Human Gene Expression v2 8x60K Microarray (Agilent Technologies). Total RNA was reverse-transcribed and labeled according to the manufacturer’s instructions. All samples were prepared and spotted in quadruplicate. Sample hybridization, microarray scanning and results extraction were performed by the GeT-Biopuces Platform in Toulouse, France.

### Microarray data analysis and gene filtration

Data analysis was performed using the AMEN suite of tools [19]. Briefly, probes showing a signal higher than a given background cutoff (median of the normalized dataset, cutoff 5.48) and at least a 2-fold change in at least one pairwise comparison were selected. To define a set of 1852 transcripts displaying significant statistical changes across comparisons, the LIMMA (linear models for microarray data) package was used (*F*-value adjusted with the false discovery rate method, *p* ≤ 0.05) [20]. The resulting probes were then partitioned into eight expression patterns (termed P1-P8) using the k-means algorithm.

### Functional data mining

The enrichment analysis module implemented in AMEN [19] was employed to identify human diseases, biological processes, molecular pathways and subcellular components significantly over-represented in each expression pattern by calculating Fisher’s exact probability using the Gaussian hypergeometric function (FDR-adjusted *p*-value ≤0.01, number of probes in a given group associated with a given annotation term ≥5).

### Regulatory network analysis

Protein-gene regulation data were downloaded from the Transcription Factor Encyclopedia database [21]. A network representation showing all known protein-gene interactions between transcripts differentially expressed in the current project was drawn using AMEN software.

### Statistical analysis

Mann-Whitney tests were performed using the BiostaTGV website (http://marne.u707.jussieu.fr/biostatgv/), and significant *p*-values were adjusted with Bonferroni correction.

## Results

### Glyceollins have anti-proliferative properties

**Fig. 1:**
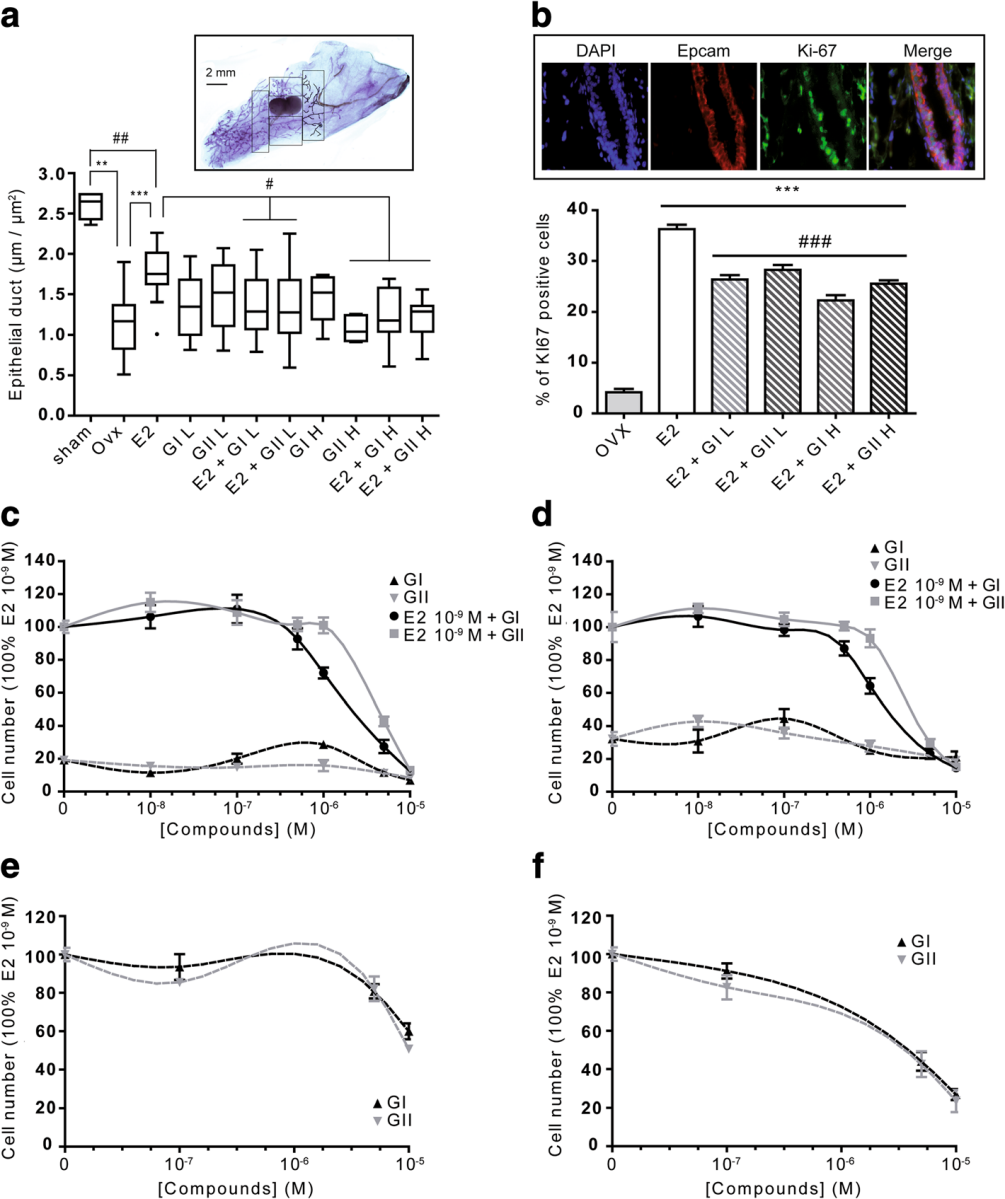
Effects of glyceollin I and II on epithelial duct growth in mouse mammary glands and on ER-positive and ER-negative breast cell lines. Mammary glands were obtained from non-ovariectomized (non-ovx) or ovariectomized (ovx) mice treated with vehicle, 10 μg/kg E2, 50 mg/kg glyceollin I or glyceollin II (GI L or GII L, respectively), or 100 mg/kg glyceollin I or glyceollin II (GI H or GII H, respectively) either alone or in combination with E2 for 72 h (**a**). Epithelial duct length was assessed by delimiting an area around the lymphatic ganglion and measuring the length of this area using FIJI software (Cartridge). The results are represented in box-and-whisker plots, where the top and the bottom of the box correspond to the 75th and the 25th percentile, respectively. The horizontal bar in the box is the median, and the points outside the box correspond to extreme values. The results are expressed in μm/μm^2^ +/− SEM and were obtained from four independent experiments with at least 5 mice per group. *p-value <0.05 and ***p-value <0.001 with a Mann-Whitney test followed by Bonferroni correction for comparisons of ovx vs treated mice. #*p*-value <0.05, ##*p*-value <0.01 and ###*p*-value <0.001 with a Mann-Whitney test followed by Bonferroni correction for comparisons of E2 vs the other treatments. The anti-proliferative effect of glyceollin was assessed using Ki-67 and Epcam immunostaining of mammary gland tissue (**b** and cartridge). The immunostaining was performed on frozen slices of mammary glands around the lymphatic ganglion for ovx and E2-treated mice in combination with the two glyceollin concentrations. Then, the number of Ki-67-positive epithelial cells was determined with FIJI software. The results are expressed as the percentage of Ki-67-positive epithelial cells and represent the mean +/− SEM of three independent mice per treatment. ***p-value <0.001 with a Mann-Whitney test followed by Bonferroni correction for comparisons of ovx vs treated mice. ###*p*-value <0.001 with a Mann-Whitney test followed by Bonferroni correction for comparisons of E2 vs the other treatments. The effects of glyceollin on cell proliferation were also assessed in the ER-positive breast cell lines MCF-7 (**c**), T47D (**d**), and HC-11 (**e**) and the ER-negative breast cell line MCF10A (**f**). Cells were grown for 6 days with or without 10^−9^ M estradiol (E2) in the presence or absence of different concentrations of glyceollins. The numbers of cells measured in the presence of glyceollin I (black hatched line) or II (grey hatched line), either alone or in combination with E2 (full line), are expressed as percentages of E2-treated cells (**c** and **d**) or untreated control cells (**e** and **f**). The results are presented as the mean +/− SEM of four independent experiments

To test the in vivo effects of synthetic glyceollins I and II on mammary gland growth and uterotrophy, we first compared the epithelial duct length (Fig. 1a) and the uterine weight (Additional file 1: Figure S1a) from controls, E2-exposed and glyceollin-exposed mice. We used ovx animals at 4.5 weeks in age, which allowed us to work with animals possessing estrogen-sensitive tissues but having never been under estrogenic influence since birth. This ensured reduction of the endogenous hormonal background, low baseline uterine weights and a maximum range of response to administered estrogens. This also allowed efficient monitoring of antiestrogenic effects after exposure to estrogens [22]. As controls, ovx mice were injected daily for three days with vehicle or 10 μg/kg E2. For the treatment groups, ovx mice were injected with 50 mg/kg glyceollin I (GI L) or II (GII L) or with 100 mg/kg GI H or GII H either alone or in combination with E2. Epithelial duct length was measured around the lymphatic ganglion on a surface representing nearly 25% of the total surface of the mammary gland (Fig. 1a cartridge). In the ovx mice, the length of the epithelial duct was markedly reduced (median, 1.17 μm/μm^2^) compared to non-ovx mice (median, 2.65 μm/μm^2^). E2 treatment partially restored the length and the ramification of the epithelial duct compared to the non-ovx animals and significantly increased them compared to the ovx mice (median, 1.75 μm/μm^2^, *p* < 0.001). Although treatment with glyceollin I or II alone seemed to enhance epithelial duct proliferation at a low dose (GI L and GII L) (median, 1.35 μm/μm^2^ and 1.52 μm/μm^2^, respectively) no significant differences were found between the ovx vehicle- and E2-treated groups. In fact, epithelial duct length in the glyceollin-treated mice varied widely, ranging from 0.805 μm/μm^2^ to 2.07 μm/μm^2^. At a high dose (GI H), glyceollin I seemed to enhance the growth of the epithelial duct (median, 1.52 μm/μm^2^). Unlike glyceollin I, high-dose glyceollin II (GII H) did not exert any estrogenic activity on the growth of galactophore ducts (median, 1.04 μm/μm^2^), which was significantly less than that in the E2-treated mice (*p* < 0.01). In contrast, glyceollins exhibited antiestrogenic activity when combined with E2. As shown in Fig. 1a, co-treatment with E2 and glyceollin I or II significantly reduced epithelial duct length (median, 1.29 μm/μm^2^ and 1.28 μm/μm^2^, respectively, *p* < 0.05 at the low dose and median, 1.18 μm/μm^2^ and 1.29 μm/μm^2^, respectively, *p* < 0.001, at the high dose) compared to the E2-treated mice. In contrast, neither glyceollin showed significant estrogenic or antiestrogenic effects on uterine weight (Additional file 1: Figure S1a). To verify that the differences observed in epithelial duct length between the E2 treatment and the combination treatment of E2 with the two doses of glyceollin I and II were linked to proliferation, Ki-67 immunostaining was performed (Fig. 1b). Three animals were analyzed from the ovx group and for each treatment group. The immunostaining was performed on slices taken from around the lymphatic ganglion, and the total number of epithelial duct cells was counted. Ki-67 immunostaining colocalized with Epcam immunostaining suggesting that Ki-67 expression is restrained to mammary epithelial cells (Fig. 1b cartridge). In ovx mice, the percentage of Ki-67-positive cells was very low at 4%. As described above, E2 treatment, either with or without concomitant glyceollin treatment, significantly increased (*p* < 0.001) the percentage of Ki-67-positive cells compared to the ovx group. However, at the two doses tested, the glyceollins significantly reduced the number of Ki-67-positive cells compared to the E2 treatment alone.

We next tested the in vitro effects of glyceollin I and II on the growth of ER-positive breast cell lines (MCF-7, T47D and HC-11) and an ER-negative nonmalignant breast cell line (MCF10-A). The cells were treated with vehicle and different doses of glyceollin I and II either alone or in combination with E2 for six days. As the proliferation of ER-positive cells is controlled by estrogens, the MCF-7 and T47D cell lines were also treated with 10^−9^ M E2, which respectively led to 5- and 3.5-fold increases in cell number (Fig. 1c and d). Interestingly, when cells were treated with E2 in combination with glyceollin I or II, a significant anti-proliferative effect was observed for a doses of 10^−6^ M glyceollin I and 5 × 10^−6^ M glyceollin II compared to the E2-treated cells. Thus, glyceollin I showed stronger anti-proliferative effects than glyceollin II. However, globally, glyceollin treatment alone did not augment cell number, except for glyceollin I at a dose of 10^−6^ M, which showed a very low proliferative effect in MCF-7 cells. It should also be noted that both glyceollins showed a weak but significant anti-proliferative effect at a dose of 10^−5^ M compared to the vehicle-treated control cells. E2 did not have any effect on the proliferation of the mouse mammary gland cell line HC-11 (data not shown). However, the glyceollins showed a strong anti-proliferative effect that was statistically significant starting from 5 × 10^−6^ M (Fig. 1e). Unlike the other ER-positive cell lines used above, glyceollin I and II showed the same dose effect. Because HC-11 cells require EGF and insulin for growth, it is possible that the glyceollins affected the signaling pathways involved by these two growth factors. To assess the precise role of the ER in this phenotype, ER-negative MCF10-A breast cancer cells were treated with different doses of glyceollin I and II for six days (Fig. 1f). A significant decrease in proliferation was observed with both glyceollins at 10^−5^ M compared to vehicle-treated control cells. However, the decrease was less evident in these cells compared to the MCF-7 or T47D cell lines. Altogether, these data show that the anti-proliferative effects of glyceollins are primarily produced by the ER but also occur through other ER-independent pathways. To establish whether the glyceollins are cytostatic or cytotoxic, cell cycle and apoptosis were assessed in MCF-7 cells after treatment with 10^−5^ M glyceollins I or II either alone or in combination with E2 (Additional files 1: Figure S1b and c, and 2 respectively). Cell cycle was analyzed by flow cytometry and showed that E2 induced MCF-7 cells to enter the cell cycle by significantly increasing the percentage of cells in S and G2/M phases compared to control cells. In contrast, glyceollins I and II only weakly induced cells to enter S phase and blocked their passage to G2/M phase. In combination with E2, glyceollins I and II reduced cell entry into S phase compared to treatment with E2 alone, but the combination clearly blocked cells from entering G2/M phase. Apoptosis was analyzed by TUNEL assay. As described in numerous previous studies, E2 reduced the percentage of apoptotic cells. In contrast, 10^−5^ M glyceollin, whether alone or in combination with E2, did not significantly increase the percentage of apoptotic cells. Thus, glyceollins appear to be more cytostatic than cytotoxic.

### Glyceollins interact with the ER

**Fig. 2:**
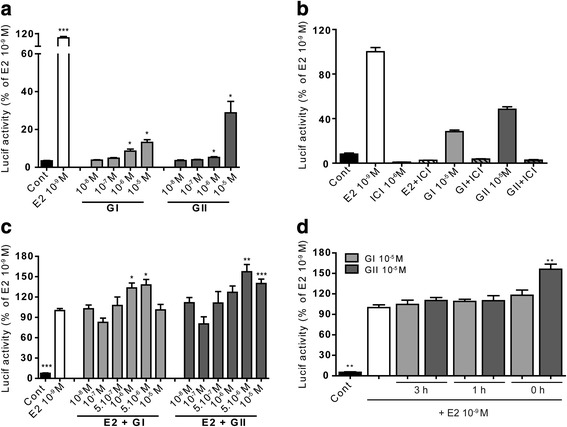
Effect of glyceollins I and II on ER activation by luciferase assay. MCF-7 cells were transfected with an ERE-TK-luciferase reporter plasmid and a CMV-β-galactosidase plasmid as a control of transfection efficiency. The cells were then treated with 10^−9^ M E2 (white) and different doses of glyceollin I (light grey) or II (hard grey) either alone (**a**), in combination with ICI_182,780_ (**b**) or in combination with E2 (**c**). Moreover, cells were pretreated with 10^−5^ M GI or GII at different time indicated (3 h, 1 h and 0 h) before 10^−9^ M E2 adding (**d**). The results are expressed as the percentage of luciferase activity obtained with E2 treatment and are represented as the mean +/− SEM of four independent experiments. *p-value <0.05, **p-value <0.01 and ***p-value <0.001 with a Mann-Whitney test followed by Bonferroni correction for comparisons of the control vs the treatments (**a**) or for treatment with E2 vs the other compounds (**c** and **d**)

To verify ER activation by glyceollins, MCF-7 cells were transfected with an ERE-TK-luciferase reporter gene containing an ERE that has been classically used to assay the estrogenic potencies of xenoestrogens (Fig. 2). The cells were treated with 10^−9^ M E2, which served as a positive control, and different concentrations of glyceollin I or II with or without 10^−6^ M ICI_182,780_ (Fig. 2a and b). As shown in Fig. 2a, both glyceollins weakly activated the ER, as a significant increase in luciferase activity was observed in cells treated with 10^−6^ M and 10^−5^ M glyceollin I or II compared to cells treated with solvent (*p* < 0.05). Nevertheless, even if these glyceollin concentrations induced expression of the luciferase reporter gene, they did not reach the maximal transactivation efficiency observed with 10^−9^ M E2. Indeed, glyceollin I and II showed approximately 7.5- and 4-fold less induction, respectively, than that obtained with E2. This activation was clearly linked to ER as shown in Fig. 2b where a co-treatment with ER-inhibitor, ICI_182,780_, abolished the increase of luciferase activity. Surprisingly, when MCF-7 cells were treated with 10^−9^ M E2 and different doses of glyceollin I or II (Fig. 2c), neither glyceollin I nor glyceollin II had an antiestrogenic effect on E2 induction. In contrast, it appears that the glyceollins acted additively with E2 to significantly increase luciferase activity; these increases were observed with 10^−6^ M and 5 × 10^−6^ M glyceollin I (*p* < 0.05) and with 5 × 10^−6^ M and 10^−5^ M glyceollin II (*p* < 0.01 and *p* < 0.001, respectively). In a recent study [23], it was shown an antagonist effect of glyceollins on ER activation in presence of E2 when cells were pretreated 1 h by glyceollins before E2 adding. Thus, we have pretreated cells with 10^−5^ M glyceollin I or II for 1 h or 3 h before adding 10^−9^ M E2 (Fig. 2d). Neither 3 h nor 1 h of pretreatment with GI affected E2-induction of luciferase activity. Like for GI, GII pretreatment did not antagonize E2 effect but the additive effect observed in absence of pretreatment was abolished.

### Glyceollins modulate E2-related gene expression

**Fig. 3:**
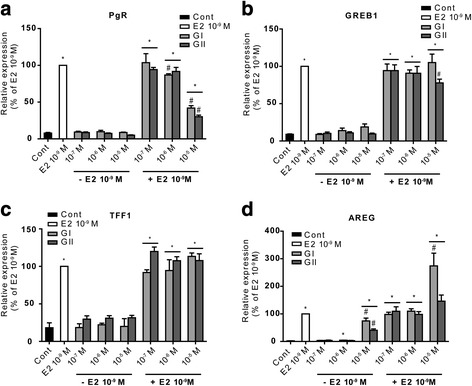
Effects of glyceollins I and II on the expression of endogenous E2 target genes. MCF-7 cells were treated with different doses of glyceollin I (white) or II (black) with or without 10^−9^ M E2. The relative expression of PgR (**a**), GREB1 (**b**), TFF1 (**c**) and AREG (**d**) was assessed by real-time PCR and normalized to the expression of the housekeeping genes GAPDH and TBP. The results are expressed as the percentage of relative expression of each transcript obtained in the E2-treated cells and are represented as the mean +/− SEM of four independent experiments. *p-value <0.05, **p-value <0.01 and ***p-value <0.001 with a Mann-Whitney test followed by Bonferroni correction for comparisons of the control vs the treatments. #*p*-value <0.05 with a Mann-Whitney test followed by Bonferroni correction for comparisons of the E2 treatment vs the other treatments

We also performed real-time PCR to evaluate the effects of glyceollins on different endogenous E2-dependent genes in MCF-7 cells. As shown in Fig. 3, expression levels of the progesterone receptor (PgR) (Fig. 3a), growth regulation by estrogen in breast cancer 1 (GREB1) (Fig. 3b), trefoil factor 1 (TFF1, also known as pS2) (Fig. 3c) and amphiregulin (AREG) (Fig. 3d) were measured after 24 h of treatment with 10^−9^ M E2 and different doses of glyceollin I or II either alone or in combination with E2. Different profiles were observed depending on the considered gene. Concerning PgR expression, the glyceollins did not show any effects when used alone. When cells were co-treated with E2 and glyceollins, a 50% decrease in PgR expression was observed with 10^−5^ M glyceollins compared to that obtained in cells treated with E2 alone. Concerning GREB1 expression, we observed a 2-fold increase in expression with glyceollin I treatment compared to the control cells. Conversely, glyceollin II did not show any effects. Co-treatment of cells with E2 and glyceollins did not considerably affect E2-mediated induction of GREB1 expression, except for a slight repression with 10^−5^ M glyceollin II. For TFF1, we observed a similar profile to that obtained with GREB1: neither glyceollin I nor glyceollin II significantly affected TFF1 gene expression regardless of the presence of E2. A third type of expression profile was obtained with the AREG gene, which is expressed at very low basal levels in MCF-7 cells. E2 treatment strongly induced AREG gene expression, increasing it by approximately 40-fold. Surprisingly, 10^−5^ M glyceollin I or II alone also induced AREG gene expression. In addition, at 10^−5^ M, both glyceollins showed an additive effect with E2. To assess if the difference observed in the transcription of these genes could be link to the recruitment of ERα on ERE, chromatin immunoprecipitation (ChIP) experiments were performed on GREB1 and PgR gene promoters Additional file 2. As shown in the Additional file 3: Figure S2, E2-treatment increased ERα recruitment on GREB1 promoter and PgR enhancer about 3 and 10 times, respectively. MCF-7 cell treated with GI or GII alone showed a slight or no effect on ERα recruitment on GREB1 and PgR promoter, respectively. However, when cells were co-treated with E2 and glyceollins, ERα recruitment on GREB1 promoter was not modified whereas it was significantly reduced on PgR enhancer. Together, these results are in good agreement with the expression profiles of these genes observed from the Q-PCR data (Fig. 3a and b).

### Genome-wide analysis of glyceollin’s effects

**Fig. 4:**
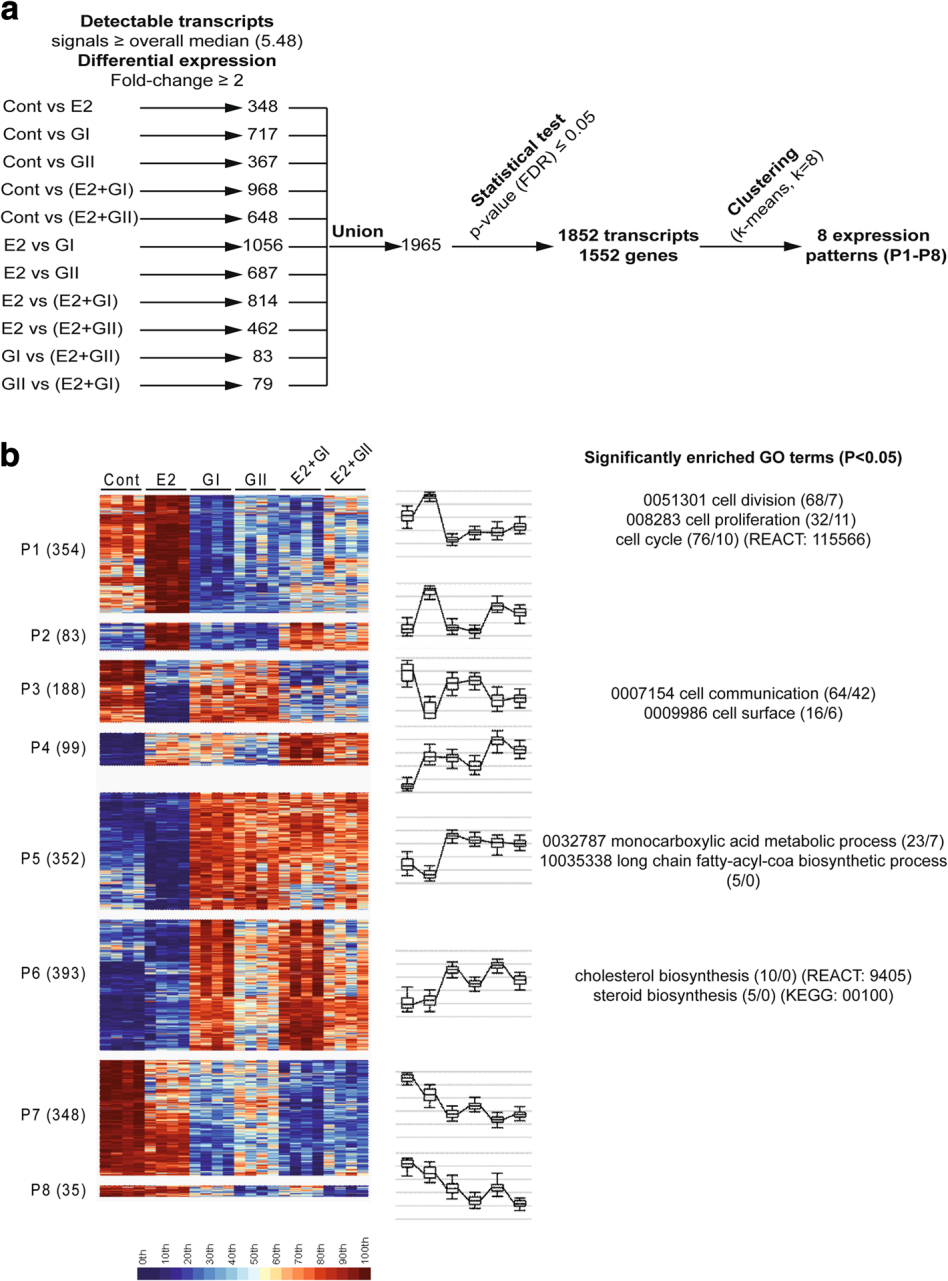
Transcriptomic analysis, selection and clustering of differentially expressed genes. MCF-7 cells were treated with vehicle, 10^−9^ M E2, 10^−5^ M glyceollin I and II, or a combination of E2 and each of the glyceollins. Total RNA was extracted, reverse-transcribed, labeled and spotted onto a DNA chip. To select differentially expressed genes, all pairwise conditions were performed. All intensity signals above the overall median and with a fold change ≥2 were selected from each comparison. Then, the selected transcripts were joined and submitted to LIMMA tests. Only transcripts with a *p*-value ≤0.05 were selected, for a total of 1550 genes (**a**). These genes were clustered into eight different expression patterns (P1-P8) (**b**). Each pattern shows the number of genes in parentheses. The conditions are classified according to control, E2, glyceollin I, glyceollin II, E2 and glyceollin I, and E2 and glyceollin II. The most relevant GO terms for each pattern are indicated at the right side of the panel. For example, we found a significant enrichment of genes associated with cell division in P1 (68 genes, 7 expected by chance), as indicated (68/7) in the figure

To more precisely understand the actions of glyceollins and to particularly assess how glyceollins affect E2 gene regulation, we performed transcriptomic analysis of MCF-7 cells treated with glyceollins either alone or in combination with E2. The method used to select differentially expressed genes is described in Fig. 4a and in materials and methods. After this initial sorting, all the selected transcripts were combined, for a total of 1965 transcripts. Then, LIMMA tests were applied with a *p*-value ≤0.05, which led to the selection of 1852 transcripts representing 1550 genes. These differentially expressed genes were clustered, and 8 expression patterns emerged (P1-P8) (Fig. 4b). All the genes from the different patterns are referenced in Additional file 4: Table S1. P1, P2, P3 and P4 corresponded to estrogen-regulated genes that were upregulated (P1, P2 and P4) or downregulated (P3). In these groups, the glyceollins exerted different effects. Glyceollin I and glyceollin II both exerted antiestrogenic activity in P1 and to a lesser extent in P2 when cells were co-treated with E2. Moreover, in P1, glyceollin reduced gene expression independently of E2 co-treatment. P4 contained genes that were induced by E2 and also by glyceollin I or II. Interestingly, when cells were co-treated with E2 and the glyceollins, an additive effect was observed between E2 and glyceollin on the induction of P4 genes. P3 corresponded to genes repressed by E2 and to a lesser extent by glyceollin I or II. In co-treated cells, the glyceollins partially restored the expression of these genes compared to E2 treatment alone. P5, P6, P7 and P8 corresponded to genes unaffected or slightly affected by E2 but regulated by the glyceollins. P5 and P6 included genes upregulated by glyceollin I or II. The difference between these patterns resided in the effect of glyceollin II on gene induction, which was globally less important in P6. P7 and P8 corresponded to genes downregulated by both glyceollins. The difference between these patterns was the effect of glyceollin II, which was a less powerful inhibitor in P7. Then, differentially expressed genes from each pattern were subjected to GO and pathway analysis (Fig. 4b and Additional file 5: Figure S3). Fig. 4b shows the most relevant significant biological processes, cellular components and pathways for patterns P1, P3, P5 and P6. For each term, the number of genes associated with the term is indicated and compared to the number of genes expected by chance. Interestingly, P1 essentially included genes linked to the cell cycle (68 genes were associated, 7 genes were expected by chance; 68/7) and cell proliferation (32/11). The effects of the glyceollins on the genes from this group could partially explain the anti-proliferative action of these compounds. In P3, GO terms related to cell communication (64/42), cell surface (16/6) and secretion pathways prevailed. In P5, as described previously [24], genes linked to monocarboxylic acid metabolism (23/7) and long fatty-acyl-CoA biosynthesis (5/0) were found. Finally, genes associated with cholesterol (10/0) and steroid biosynthesis (5/0) were found in P6. A Venn diagram is shown in Additional file 6: Figure S4.

**Fig. 5:**
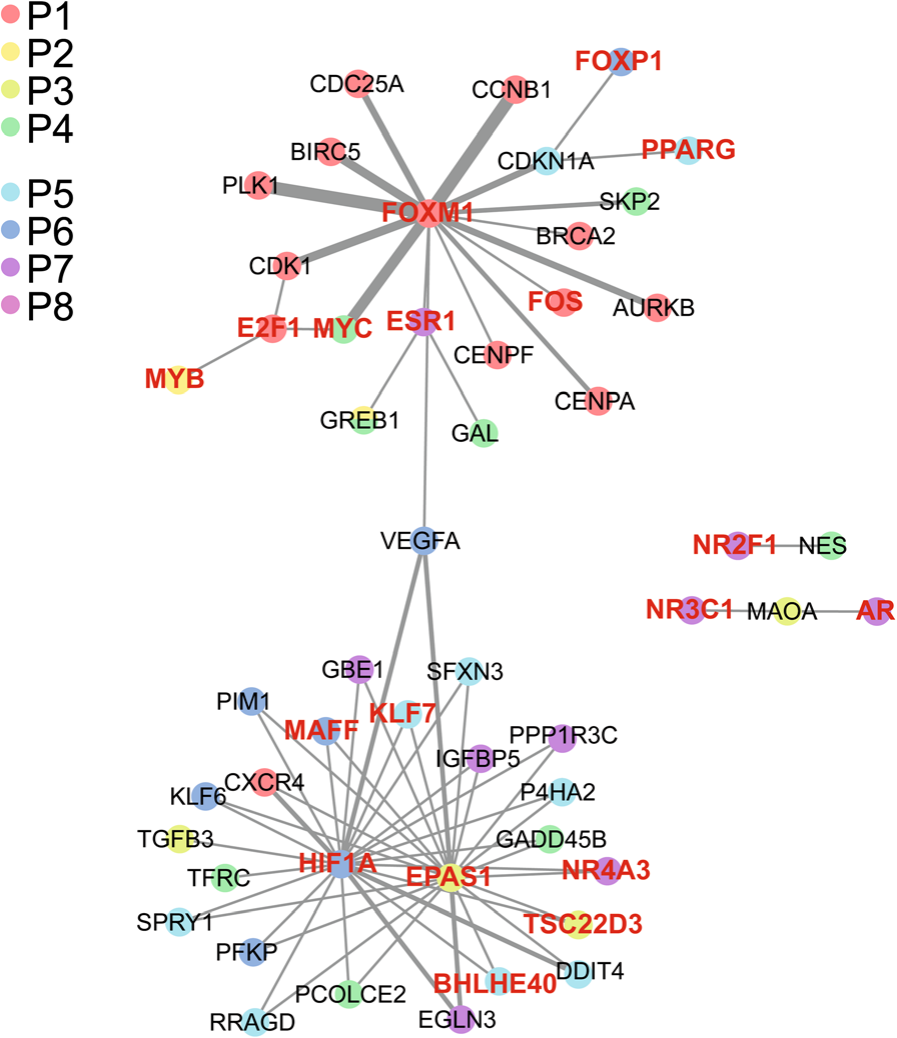
Gene regulatory network built from transcript profiling data and regulation data. Each gene/protein is represented by a node color-coded according to the related expression pattern. The edges between the nodes correspond to protein/DNA interactions, and the thickness of these strings is dependent on the number of publications describing the interaction. Genes coding for transcription factors are shown in red

Regulatory network analysis combining the differentially expressed gene data as well as the protein-gene interaction data showed a small network with two principal communities represented by three TFs, FOXM1, HIF1α and EPAS1/HIF2α (Fig. 5). In the FOXM1 group, genes from P1 were overrepresented, as shown by light red circles, whereas in the community with HIFα and EPAS1/HIF2α, E2-independent genes from P5, P6 and P7 were more important. However, glyceollins might regulate cell functions through two pathways, one involving ER and FOXM1 and the other involving the HIF pathway.

### Genes linked to proliferation and growth arrest were differentially expressed

**Fig. 6:**
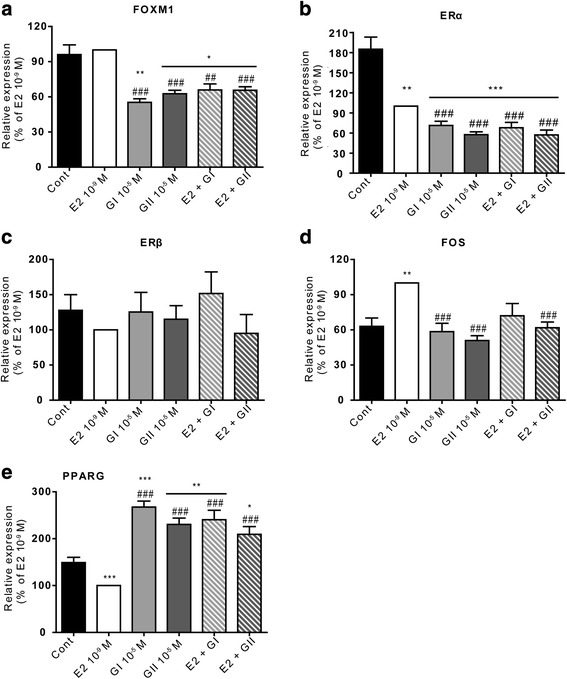
Validation of differentially expressed genes corresponding to the FOXM1 community. MCF-7 cells were treated with vehicle (black), 10^−9^ M E2 (white), 10^−5^ M glyceollin I (light grey) and II (hard grey), or a combination of E2 and each of the glyceollins (hatched squares). The relative expression of FOXM1 (**a**), ERα (**b**), ERβ (**c**), FOS (**d**) and PPARG (**e**) was assessed by real-time PCR and normalized to the expression of the housekeeping genes GAPDH and TBP. The results are expressed as the percentage of relative expression of each transcript obtained in E2-treated cells and are represented as the mean +/− SEM of 10 independent experiments. *p-value <0.05, **p-value <0.01 and ***p-value <0.001 with a Mann-Whitney test followed by Bonferroni correction for comparisons of the control vs the treatments. ##*p*-value <0.01 and ###*p*-value <0.001 with a Mann-Whitney test followed by Bonferroni correction for comparisons of E2 vs the other treatments

As described above, glyceollins I and II affected FOXM1 expression and, consequently, downstream genes involved in G2/M transition and mitosis, such as CENPA, CENPF, AURKB and PLK1 [25, 26] (Fig. 6 and Additional file 4: Table S1). However, to validate the results from our transcriptomic analysis, genes not involved in G2/M transition but linked to FOXM1 were chosen (Fig. 6). Expression levels of FOXM1, ERα, ERβ, FOS and PPARγ were assessed by real-time PCR. Cells were treated under the same conditions as those used for the transcriptomic analysis, and the results are expressed as the percentage of normalized expression following E2 treatment. FOXM1 (Fig. 6a) was not induced by E2 at 10^−9^ M but was significantly reduced by glyceollin I and II both in the presence (*p* < 0.01 and *p* < 0.001, respectively) and absence of E2 (*p* < 0.001). ERα was affected and linked to FOXM1 in our network analysis (Fig. 5). To further understand the antiestrogenic properties of the glyceollins, ERα expression was also measured. ERα levels were significantly reduced (*p* < 0.01) in E2-treated cells compared to control cells. Glyceollin I and II were both strong inhibitors of ERα expression. As shown in Fig. 6b, ERα expression was significantly reduced in glyceollin I- and glyceollin II-treated cells compared to E2-treated cells regardless of whether they were treated with (*p* < 0.01) or without E2 (*p* < 0.01 and *p* < 0.001, respectively). In contrast, ERβ expression was not affected by either E2 or glyceollin I or II (Fig. 6c). FOS is involved in tumorigenesis and ER signaling. FOS expression was significantly induced (*p* < 0.01) by E2 treatment but not by treatments with glyceollin I or II. Moreover, glyceollin II significantly inhibited the E2-mediated induction of FOS expression (*p* < 0.001) (Fig. 6d). Glyceollin I also tended to inhibit this E2-mediated induction, but the effect was not statistically significant. Finally, as glyceollins have been shown to regulate lipid metabolism [24], we also measured the expression of PPARγ, a member of the FOXM1 community. As shown in Fig. 6e, PPARγ expression was significantly downregulated in E2-treated cells compared to controls (*p* < 0.001), whereas it was significantly upregulated when cells were treated with glyceollin I or II either in the presence or absence of E2 (*p* < 0.001) compared to E2 alone.

**Fig. 7:**
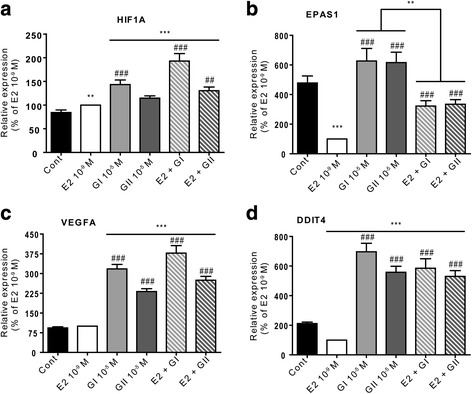
Validation of differentially expressed genes corresponding to the HIF1α/EPAS1 community. MCF-7 cells were treated with vehicle (black), 10^−9^ M E2 (white), 10^−5^ M glyceollin I (light grey) and II (hard grey), or a combination of E2 and each of the glyceollins (hatched squares). The relative expression of HIF1α (**a**), EPAS1 (**b**), VEGFA (**c**) and DDIT4 (**d**) was assessed by real-time PCR and normalized to the expression of the housekeeping genes GAPDH and TBP. The results are expressed as the percentage of relative expression of each transcript obtained in E2-treated cells and are represented as the mean +/− SEM of 10 independent experiments. **p-value <0.01 and ***p-value <0.001 with a Mann-Whitney test followed by Bonferroni correction for comparisons of the control vs the treatments. ##*p*-value <0.01 and ###*p*-value <0.001 with a Mann-Whitney test followed by Bonferroni correction for comparisons of E2 vs the other treatments

Next, the expression of genes linked to the hub created by HIF1α and EPAS1/HIF2α was also validated by real-time PCR (Fig. 7). HIF1α was slightly but significantly induced following E2 treatment compared to the control (*p* < 0.01). Unlike glyceollin II, glyceollin I was 1.5-fold more potent in inducing HIF1α expression than E2 (*p* < 0.001). In addition, when cells were co-treated with E2 and glyceollin I, we observed a synergistic effect resulting in 2-fold higher E2-mediated induction of HIF1α expression (*p* < 0.001). Cells co-treated with E2 and glyceollin II showed significantly induced HIF1α expression (*p* < 0.01) compared to those treated with E2 alone (Fig. 7a). EGLN3, part of the HIF1α community, encodes a protein involved in the degradation of HIF1α under normoxic conditions and was downregulated by glyceollin I and II (Additional file 4: Table S1). Furthermore, the expression of EPAS1/HIF2α was strongly repressed by E2 by 5-fold (*p* < 0.001) compared to the control. Glyceollin I and II did not affect EPAS1/HIF2α expression compared to the control. In co-treated cells, glyceollin I and II partially restored the expression of this gene (Fig. 7b). A classical target of HIF1α is VEGFA. Therefore, it was not surprising that this gene emerged in our analysis. Compared to control cells, VEGFA expression was not induced by E2 treatment, but it was induced by treatments with glyceollin I and II (3.5-fold and 2.5-fold, respectively; *p* < 0.001) independently of E2 co-treatment (Fig. 7c). Finally, it is interesting to note that our network analysis identified a few genes associated with stress response and growth arrest, such as GADD45B or DDIT4 L (REDD2) and DDIT4 (REDD1). This last gene was chosen to validate the transcriptomic analysis results. DDIT4 expression was significantly reduced (*p* < 0.001) following E2 treatment compared to the control and was significantly induced by glyceollin I and II (3.5-fold and 3-fold, respectively; *p* < 0.001). In co-treated cells, E2 had a slight impact on glyceollin I and II-mediated induction of DDIT4 (6-fold and 5.5-fold, respectively, compared to E2 alone; *p* < 0.001).

**Fig. 8:**
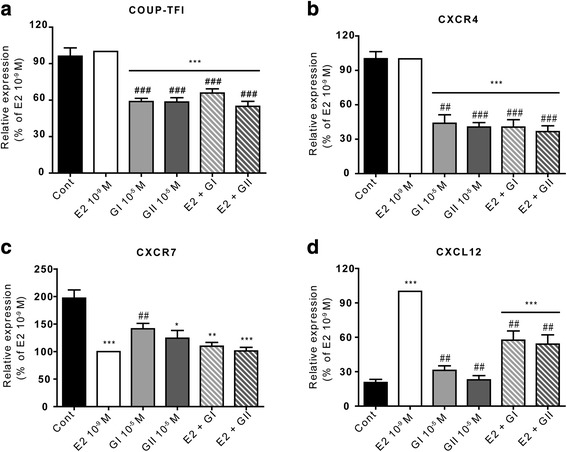
Validation of differentially expressed genes corresponding to the CXCL12/CXCR4/CXCR7 axis. MCF-7 cells were treated with vehicle (black), 10^−9^ M E2 (white), 10^−5^ M glyceollin I (light grey) and II (hard grey), or a combination of E2 and each of the glyceollins (hatched squares). The relative expression of COUP-TFI (**a**), CXCR4 (**b**), CXCR7 (**c**) and CXCL12 (**d**) was assessed by real-time PCR and normalized to the expression of the housekeeping genes GAPDH and TBP. The results are expressed as the percentage of relative expression of each transcript obtained in E2-treated cells and are represented as the mean +/− SEM of 10 independent experiments. *p-value <0.05, **p-value <0.01 and ***p-value <0.001 with a Mann-Whitney test followed by Bonferroni correction for comparisons of the control vs the treatments. ##*p*-value <0.01 and ###*p*-value <0.001 with a Mann-Whitney test followed by Bonferroni correction for comparisons of E2 vs the other treatments

The final pathway from the transcriptomic data is the CXCR4/CXCR7/CXCL12 axis, which is associated with the orphan receptor COUP-TFI (Fig. 8). In previous studies [27, 28], we have reported on the importance of the role of COUP-TFI in this signaling axis, which modulates the proliferation and migration of breast cancer cells. Indeed, breast tumor aggressiveness has been associated with increased COUP-TFI expression, decreased CXCL12 and CXCR7 expression, and increased CXCR4 expression. However, it is interesting to note that treatment with glyceollin I or II significantly reduced the expression of COUP-TFI and CXCR4 by 2-fold (*p* < 0.001 and *p* < 0.01, respectively, compared to E2) with or without E2 co-treatment (Fig. 8a and b). As previously described, E2 significantly decreased CXCR7 expression compared to the control (*p* < 0.001) (Fig. 8c). Glyceollin I and II also repressed CXCR7 expression; however, glyceollin I was significantly less potent than glyceollin II. Furthermore, neither of the glyceollins had an effect on E2-mediated repression of CXCR7 expression when used in co-treatments. E2 increased the expression of CXCL12 by approximately 5-fold, whereas glyceollin I and II did not modify CXCL12 expression compared to the control. In contrast, both glyceollins markedly reduced the E2-medited induction of CXCL12 expression (Fig. 8d).

## Discussion

Estrogens are involved in multiple physiological processes and act on various tissues. In particular, they participate in the development and maintain the function of reproductive organs such as the gonads or the mammary gland through their binding to ERα and ERβ. ERα is the major isoform in the mammary gland and has a role in epithelial duct proliferation and differentiation [29]. E2 also promotes cell survival, and due to its proliferative effect, this hormone has been linked to breast cancer [3]. Many natural and synthetic chemicals in the environment have been reported to exhibit hormonal activity, particularly estrogenic potency [30]. This is the case for the well-known compound bisphenol A, which has well-documented effects on breast cancer [31]. Phytoestrogens, which are estrogenic compounds from plants, are also found in food, particularly in soy, and have been reported to decrease the risk of breast cancer at high doses [32, 33]. Among the phytoestrogens, glyceollins emerged as promising compounds in the 2000s.

A previous study from Burow et al. [11] reported that glyceollins bind the ER and act as antiestrogenic compounds by inhibiting cell proliferation. However, the detailed molecular mechanisms driving the anti-proliferative actions of these phytochemicals remain elusive and appear to be more complex than those based only on ER interactions [14]. In the present work, we utilized synthetic glyceollins I and II to better delineate the modes of action exhibited by these compounds. We showed that glyceollin I and glyceollin II inhibit the trophic action of E2 in vivo in mouse mammary glands, but not in uteri. Our data differ from those obtained by Salvo et al., who reported that natural glyceollins antagonize the trophic effects of E2 in uteri [13]. One explanation for these different observations regarding uterotrophy is that the referenced study used nude mice that were treated daily with the natural glyceollins for twenty days, whereas our current study treated mice for only three days. Nevertheless, our data clearly show that mammary epithelium growth is not influenced by glyceollins when they are administered alone; however, when administered together with E2, they are capable of inhibiting the stimulatory effect of E2 on ductal epithelium growth. It should be noted that since proliferation is partially blocked by glyceollins during ductal elongation, further in vivo experiments, such as TUNEL assays, would be required to determine other mechanisms of action by which glyceollins may block E2-mediated ductal elongation. However, to the best of our knowledge, this is the first in vivo study showing the anti-proliferative effect of glyceollins on epithelial ductal extension. In this way, glyceollins appear to be selective estrogen receptor modulators (SERM). In accordance with our results, a previous study investigated the effects of various SERM such as raloxifen, on mammary gland development [34]. The authors showed that raloxifen alone induced a slight ductal tree invasion in the fat pad, whereas, in combination with conjugated estrogens, raloxifen had a clear antagonistic effect [34]. Next, we determined the in vitro effects of glyceollins on cell proliferation. Interestingly, we found that glyceollins I and II exert anti-proliferative effects in both ER-positive and ER-negative breast cancer cells in accordance with the study of Rhodes et al. [35]. This suggests that glyceollins do not act as conventional antiestrogens, such as tamoxifen, but rather act through both ER-dependent and ER-independent pathways, although the ER-dependent pathway seems to be predominant. Burow and collaborators reported that glyceollins could antagonize the E2-mediated stimulation of an ERE-luciferase reporter plasmid [11, 12, 15]. Although this was not observed in our study, the use of different ERE sequences and cell lines and differences in the duration of the treatment could account for this discrepancy. For example, in our experiments, an ERE-luciferase reporter plasmid was used that contains only a single ERE sequence upstream of the luciferase gene, whereas Burow and collaborators used a luciferase reporter with two ERE motifs in addition to pre-treating cells with glyceollins before E2 was added. Based on these observations, we suggest that glyceollins may prevent cooperative effects between ER dimers on ERE sequences, which could explain the decreased luciferase activity. Nevertheless, glyceollin I and II inhibited the expression of the endogenous PgR gene induced by E2 by over 50% in MCF-7 cells.

To further explore the molecular mechanisms underlying how glyceollins exert their anti-proliferative effects, we performed transcriptomic analysis of MCF-7 breast cancer cells exposed to glyceollins and created a gene regulatory network of differentially expressed genes. This integrative genomic approach was followed by the quantification of several key genes, which allowed us to identify, for the first time, two major pathways involving the ER and FOXM1 factors and the other including the hypoxia inducible factor (HIF) family (HIF1α and EPAS1/HIF2α). The first hub highlighted in our gene regulatory network is represented by the forkhead transcription factor FOXM1 and the ER. FOXM1 is a well-known key regulator of the cell cycle and is involved in G1/S and G2/M transition [36]. Thus, the downregulation of this gene could explain the effects of glyceollins on cellular proliferation. FOXM1 gene expression involves ERα [37], and in return, expression of ERα involves FOXM1 [38]. By targeting ERα, glyceollins could affect this auto-regulatory loop. The mechanisms by which glyceollins act through ERβ are not fully defined. Competition binding assays showed that glyceollins are able to bind both ERα and ERβ with, however, a greater sensitivity of glyceollins for ERα vs. ERβ [11]. In addition, since MCF-7 cells express mainly ERα (the ratio ERα/ERβ is 8/1) [5], the effects of glyceollins are likely mediated by ERα signaling. Nevertheless, glyceollins may affect ERα/FOXM1 regulatory loop by another pathway that may potentially involve ERβ. Indeed, ERβ represses FOXM1 expression by displacing ERα from the FOXM1 promoter [39]. Our data showed that glyceollins markedly decreased ERα expression but do not affect ERβ expression in MCF-7 cells. A change in the equilibrium of the ERα / ERβ ratio could then contribute to the antiestrogenic activity exerted by the glyceollins and could reinforce the possibility of an involvement of the ERβ. In addition to the auto-regulatory loop that exists between ERα and FOXM1, decreased expression and activity of these two factors could explain the downregulation of GREB1, at least with glyceollin II. Indeed, FOXM1 and ERα co-bind DNA in breast cancer cells and modulate the expression of specific genes [40]. In the referenced work, the authors showed that FOXM1 knockout affected GREB1 expression. Therefore, one could easily hypothesize that glyceollins inhibit E2-related gene expression via this pathway. Moreover, overexpression of FOXM1 is a hallmark of many cancers and a sign of poor prognosis. In ER-positive breast cancer, overexpression of FOXM1 is associated with endocrine resistance and invasiveness because it favors the expansion of stem-like cancer cells [26]. A recent study showed that the FOXM1 cistrome is a powerful index to predict breast cancer outcomes [41]. Thus, it will be very interesting to test the plasticity of the binding interaction that exists between FOXM1 and ERα in response to glyceollin treatment.

The second hub highlighted in our gene regulatory network is centered on the HIF family. The HIF family is composed of three O_2_-regulated members (HIF1α, EPAS1/HIF2α and HIF3α) that become stabilized under hypoxic conditions. To accomplish this, they heterodimerize with the constitutively expressed HIF1β (also known as ARNT) to regulate genes necessary for adaptation to low-oxygen conditions [42]. It was surprising that the glyceollins in this study induced HIF1α expression under normoxic conditions because they have been previously described as inhibitors of this factor at both the synthesis and stability levels under hypoxic conditions [43]. Under normoxic conditions, HIF1α is controlled by numerous stimuli, including reactive oxygen species (ROS) [44]. Recently, it was shown that glyceollin at a concentration of approximately 18 μM induced ROS generation in a hepatic cell line [45]. In our experiments utilizing 10 μM glyceollin, it is possible that moderate ROS production was induced that consequently induced HIF family activity. We identified DDIT4 (also known as REDD1) and DDIT4 L (also known as REDD2), both inhibitors of mTORC1 [46], in the HIF family community. REDD1 and REDD2 are stress-responsive genes induced by different stimuli, such as DNA damage or hypoxia. mTORC1 is a member of the PI3K/AKT signaling pathway and acts downstream of AKT. It participates in protein synthesis by promoting the phosphorylation of p70S6K. Therefore, glyceollins alter the phosphorylation of p70S6K in ER-positive breast cancer [23]. Thus, inhibition of mTORC1 could be a factor involved in the anti-proliferative effects produced by glyceollins due to perturbations in the PI3K/AKT/mTOR pathway. PI3K mutations are frequently observed in ER-positive breast cancer. Many inhibitors of PI3K pathway are under clinical trials or approved as therapeutics such as everolimus which is a mTORC1 inhibitor [47]. Thus, this observation reinforces the therapeutic potential of glyceollin in ER-positive breast cancer. Moreover, another study reported that loss of the REDD1 gene leads to an increase in HIF1 level and consequently an increase in tumorigenicity. The authors also showed that REDD1 localizes to mitochondria to regulate ROS production [48]. Overall, REDD1 appears to act as a tumor suppressor that works through different levels, reinforcing the therapeutic potential of glyceollins.

Glyceollins are studied in part for their ability to inhibit E2-related gene expression [12]. Thus, we were surprised to note that, unlike the other genes that were assessed, AREG expression was induced by 10^−5^ M glyceollins, and this effect was increased by E2 co-treatment. AREG is regulated by numerous transcription factors, including the ER [49]. Recently, a role for EPAS1/HIF2α in the induction of AREG expression was described in MCF-7 cells [50]. Thus, glyceollins might induce AREG expression through ERs and EPAS1/HIF2α, which would explain the synergistic effect. Moreover, high expression of EPAS1/HIF2α, AREG and WISP2 is linked to improved survival in breast cancer [50]; glyceollin treatment does not affect EPAS1/HIF2α expression in the absence of E2 treatment and even partially restores expression in E2-treated cells. AREG and WISP2 (Fig. 3 and Additional file 4: Table S1) are overexpressed in glyceollin-treated cells.

Finally, our transcriptomic analysis identified differential effects on the expression of the orphan receptor COUP-TFI and the chemokine CXCL12, as well as its receptors CXCR4 and CXCR7, following glyceollin treatment. Considering the important role of COUP-TFI in CXCL12 expression and the importance of the CXCL12 signaling axis in tumor growth and metastasis, the effects produced by glyceollins seem very important. Indeed, the chemokine CXCL12 plays critical roles in cell migration, angiogenesis, proliferation, and survival in many types of cancer, including breast cancer, by interacting with the transmembrane receptors CXCR4 and CXCR7 [51, 52]. CXCR4 is often overexpressed in metastatic tumors and promotes the migration of invasive cells to tissues where local CXCL12 secretion is increased, such as bone, liver, brain and lung [53, 54]. We recently reported that E2 controls the activity of the CXCL12/CXCR4/CXCR7 signaling axis in breast tumor cells and influences the proliferation and migration of breast cancer cells [27, 28]. Furthermore, COUP-TFI and the CXCL12 signaling axis are dysregulated in breast tumor biopsies compared to normal epithelium. Indeed, primarily in ER-positive invasive ductal cancer, we observed significant upregulation of COUP-TFI and CXCR4 and downregulation of CXCR7 and CXCL12, and the levels of these changes showed correlations with tumor grade [28]. Downregulation of CXCL12 in cancer cells is frequently associated with promoter methylation, which encourages cells to migrate toward a CXCL12 gradient and establish metastases [55]. Interestingly, glyceollins repress the expression of CXCR4 and do not affect CXCR7. However, they exert antiestrogenic activity in E2-mediated induction of CXCL12 and they maintain the expression of this gene; thus, they might limit the metastatic potential of tumor cells. In accordance with our observation, a previous study showed that glyceollins could reverse the epithelial to mesenchymal transition of letrozole resistant cells and thus decreased their invasion and migration [56]. It would therefore be interesting to test the ability of glyceollins to limit the loss of expression of key genes in cancer and, in particular, the activation of enzymes involved in epigenetic modifications, as was previously described for genistein [57].

## Conclusion

In conclusion, glyceollins I and II did not show any effect on mouse uterotrophy, whereas they did exert antiproliferative effects on mammary gland epithelial duct growth. This antagonistic activity was confirmed in different ER-positive and ER-negative breast cell lines. Our mechanistic studies revealed that glyceollins are more cytostatic than cytotoxic. Moreover, they have some similarity to SERMs which have partial agonist and antagonist properties depending on E2-target genes. For the first time, a genome-wide microarray was performed on an ER-positive breast cell line to identify pathways involved in the anti-proliferative effects of glyceollins. We identified two major pathways, centered on FOXM1/ERα and HIF1α/HIF2α, which could explain the activity of glyceollins on ER-positive and ER-negative cell lines. These results confirm and reinforce the therapeutic potential of glyceollins for managing breast cancer.

## Additional files

Additional file 1: Figure S1. Effect of glyceollin I and II on ovariectomized mouse uterotrophy and on cell cycle and apoptosis in MCF-7 cells. Uteri were obtained from ovariectomized (ovx) or intact (non-ovx) mice treated with vehicle, 10 μg/kg E2, 50 mg/kg glyceollin I or glyceollin II (GII) (GI L or GII L), or 100 mg/kg glyceollin I or glyceollin II (GI H or GII H) either alone or in combination with E2 for 72 h (Additional file 2: Figure S1a). The mice were then sacrificed, and their uteri were removed and weighted. The results are represented in box-and-whisker plots, where the top and the bottom of the box correspond to the 75th and the 25th percentile, respectively. The horizontal bar in the box is the median, and the points outside the box correspond to extreme values. The results are expressed as relative uteri weight (g per g of body weight) and were taken from 4 independent experiments with at least 5 mice per group. ***p-value <0.001 with a Mann-Whitney test followed by Bonferroni correction for comparisons of the control vs the treatments. ##*p*-value <0.01 with a Mann-Whitney test followed by Bonferroni correction for comparisons of E2 vs the other treatments. For analyses of cell cycle (Additional file 2: Figure S1b) and apoptosis (Additional file 2: Figure S1c), cells were treated for 3 days with 10^−9^ E2 with or without 10^−5^ M glyceollin I or II. To analyze cell cycle, cells were stained with propidium iodide and subjected to flow cytometry analysis. The results are expressed as the percentages of cells in each cell cycle phase and are represented as the mean of 4 independent experiments +/− SEM. *p-value <0.05, **p-value <0.01 and ***p-value <0.001 with a Mann-Whitney test followed by Bonferroni correction for comparisons of the control vs the treatments. For analysis of apoptosis, cells were stained using a TUNEL assay, and the percentage of apoptotic cells was assessed with an Array Scan VTI. The results are expressed as the percentage of TUNEL-positive cells compared to total cells. (TIFF 2098 kb)

Additional file 2: Supplemental material. (PDF 157 kb)

Additional file 3: Figure S2. Effect of glyceollins on ERα recruitment on GREB1 promoter and PgR enhancer. MCF-7 cells were treated with vehicle (black), 10^−9^ M E2 (white), 10^−5^ M glyceollin I (light grey) and II (hard grey), or a combination of E2 and each of the glyceollins (hatched squares). The recruitment of ERα on GREB1 promoter (a) and PgR enhancer (b) was assessed by chromatin immunoprecipitation followed by real time PCR. Results are expressed in fold recruitment compared to control and are the mean of two independent experiments. (TIFF 940 kb)

Additional file 4: Table S1. All differentially expressed genes. The ID of all genes are indicated with their functions and expression levels. *Cell cycle analysis*. MCF-7 cells (100,000 cells/well) were plated in 6-well plates. After 72 h of serum and steroid deprivation, the cells were treated for 72 h with solvent as control, 10^−9^ M E2, 10^−5^ M glyceollin I or II, or a combination of E2 and glyceollin I or II. Then, the cells were trypsinized and fixed in 70% ethanol before staining with propidium iodide. The percentage of cells in each cell cycle phase was assessed by flow cytometry with a FACS Calibur (BD Biosciences). *Measurement of apoptosis* MCF-7 cells (4000 cells/well) were plated in 96-well plates. After 72 h of serum and steroid deprivation, the cells were treated for 72 h with solvent as control, 10^−9^ M E2, 10^−5^ M glyceollin I or II, or a combination of E2 and glyceollin I or II. TUNEL staining was assessed with an In Situ Cell Death Detection Kit, Fluorescein (Roche) according to the manufacturer’s instructions. The fluorescence and percentage of TUNEL-positive cells were determined with an Array Scan VTI (Thermo Fisher Scientific) on the ImPACcell platform (Rennes, France). *Chromatin Immunoprecipitation (ChIP)* MCF-7 cells (2,000,000 cells /dishes) were plated in 10 cm dishes and then deprived of steroids and serum for 72 h. The cells were treated for 1 h with 10^−9^ M E2, with 10^−5^ M GI or GII with or without 10^−9^ M E2. Then, cells were cross-linked for 10 min with 1.5% of formaldehyde (Sigma). Cells were lysed in lysis buffer (50 mM Tris-HCl, pH 8.1, 10 m M EDTA, 0.5% Empigen BB and 1% SDS). Chromatin was sonicated 10 min (15 s on/off cycles) on Bioruptor (Diagenode) at highest intensity. Soluble chromatin was diluted in IP buffer (20 mM Tris-HCl, pH 8.1, 2 mM EDTA, 0.1% Triton X-100) with 2 μg of ERα antibody (E115, Abcam) and yeast RNA as non-specific competitor and incubated overnight at 4 °C on rocking platform. Then, protein G coupled sepharose beads were added to the samples and were incubated 4 h à 4 °C. Immune complexes were washed one time in washing buffer 1 (20 mM Tris-HCl, pH 8.1, 2 mM EDTA, 150 mM NaCl, 1% Triton X-100 and 0.1% SDS), one time in washing buffer 2 (20 mM Tris-HCl, pH 8.1, 2 mM EDTA, 500 mM NaCl, 1% Triton X-100 and 0.1% SDS), one time in washing buffer 3 (10 mM Tris-HCl, pH 8.1, 1 mM EDTA, 250 mM LiCl, 1% Deoxycholate and 1% NP-40) and finally two times in washing buffer 4 (10 mM Tris-HCl, pH 8.1, 1 mM EDTA). After washing, immune complexes were extracted with 100 μl of extraction buffer (0.1 M NaHCO_3_ and 1% SDS). Cross-linking was reverse by incubation of samples overnight at 65 °C and DNA was purified using the Nucleospin Gel and PCR cleanup kit (Macherey Nagel). Enrichment analysis on the ERE proximal of GREB1 (Fwd: CACTTTGAGCAAAAGCCACA and Rev.: GACCCAGTTGCCACACTTTT) and on an enhancer 1 of PgR described in [58] was normalized using an irrelevant region on the chromosome 10 (Fwd: AGGTGACAAGCCAAGTGTCC and Rev.: GCCTGGTGGCATACTAAAGG). Analysis was performed by real time PCR on a CFX 384 apparatus (BioRad) on 2 μL of immunoprecipitation or 0.2 μL of input with 500 nM of primers and iTaq Universal SYBR Green Supermix (BioRad). (XLSX 590 kb)

Additional file 5: Figure S3. GO enrichment analysis of different treatment-related expression patterns. Eight expression patterns are matched with a selection of GO terms from the ontology “phenotypes,” “biological process,” “cellular component” and “pathways.” The numbers of genes associated with each GO term are indicated in the first column. Enrichment is indicated by bolded rectangles, where the first number indicates the number of genes found in our analysis and the second the number expected with a random list of genes. Overrepresented genes in a specific GO term are shown in red, and underrepresented genes are shown in blue. (TIFF 2724 kb)

Additional file 6: Figure S4. Venn diagram. A Venn diagram was created from the list of differentially expressed genes obtained from comparisons of the control and E2 (red), GI (yellow), GII (green), E2 + GI (blue) and E2 + GII (purple) treatments. (TIFF 3761 kb)

## Abbrevations

E2: Estradiol
ER: Estrogen receptor
ERE: Estrogen responsive element
LIMMA: Linear model for microarray data
OVX: Ovariectomized
SERM: Selective estrogen receptor modulator
