# Supplementary figures and images for: Glyceollins trigger anti-proliferative effects through estradiol-dependent and independent pathways in breast cancer cells
Source: Cell Commun Signal. 2017 Jun 30;15:26. doi: 10.1186/s12964-017-0182-1 (PMC5493871; doi:10.1186/s12964-017-0182-1)

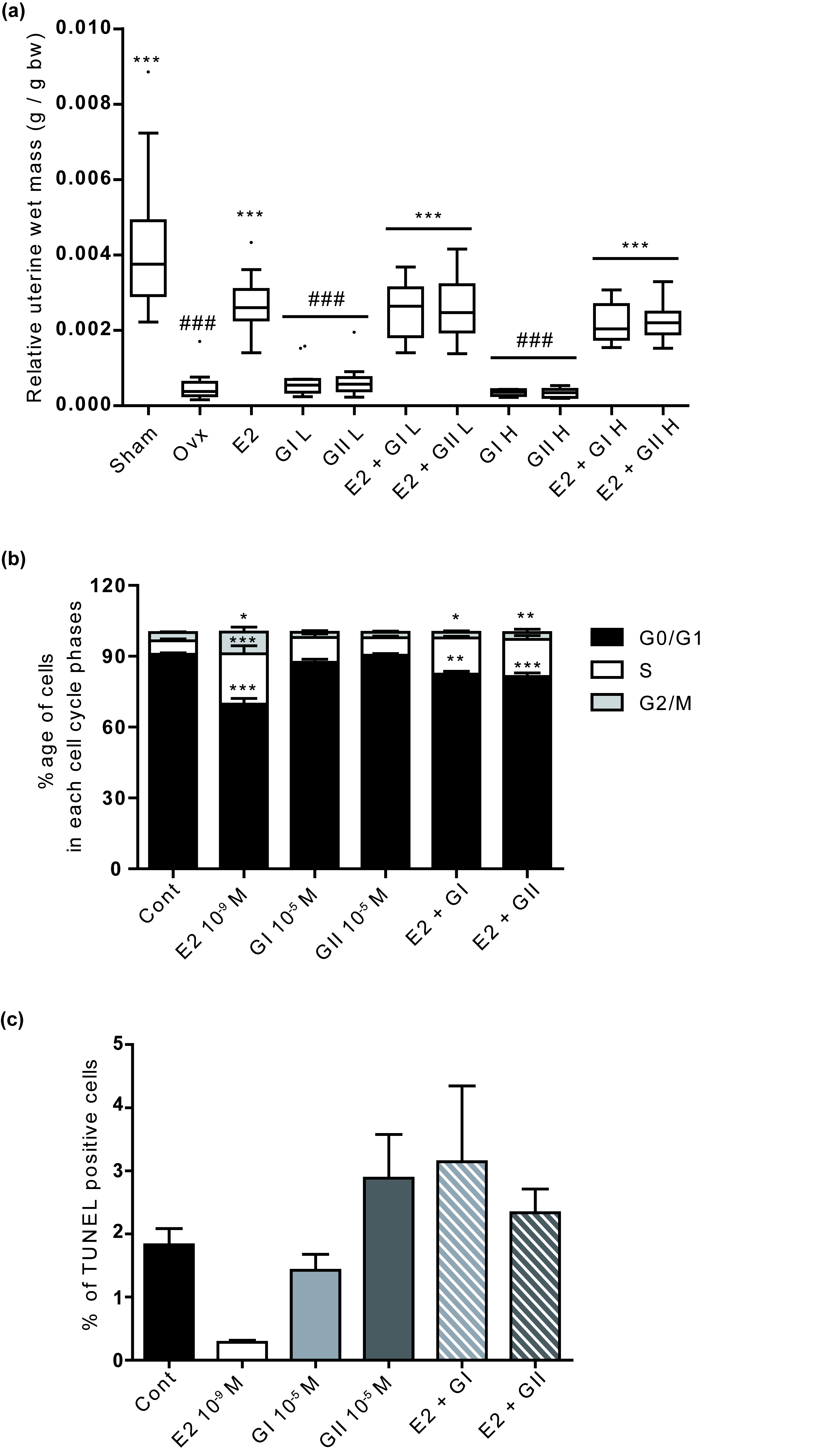

Supplement: Supplementary file 1 — Effect of glyceollin I and II on ovariectomized mouse uterotrophy and on cell cycle and apoptosis in MCF-7 cells. Uteri were obtained from ovariectomized (ovx) or intact (non-ovx) mice treated with vehicle, 10 μg/kg E2, 50 mg/kg glyceollin I or glyceollin II (GII) (GI L or GII L), or 100 mg/kg glyceollin I or glyceollin II (GI H or GII H) either alone or in combination with E2 for 72 h (Additional file 2: Figure S1a). The mice were then sacrificed, and their uteri were removed and weighted. The results are represented in box-and-whisker plots, where the top and the bottom of the box correspond to the 75th and the 25th percentile, respectively. The horizontal bar in the box is the median, and the points outside the box correspond to extreme values. The results are expressed as relative uteri weight (g per g of body weight) and were taken from 4 independent experiments with at least 5 mice per group. ***p-value <0.001 with a Mann-Whitney test followed by Bonferroni correction for comparisons of the control vs the treatments. ##p-value <0.01 with a Mann-Whitney test followed by Bonferroni correction for comparisons of E2 vs the other treatments. For analyses of cell cycle (Additional file 2: Figure S1b) and apoptosis (Additional file 2: Figure S1c), cells were treated for 3 days with 10−9 E2 with or without 10−5 M glyceollin I or II. To analyze cell cycle, cells were stained with propidium iodide and subjected to flow cytometry analysis. The results are expressed as the percentages of cells in each cell cycle phase and are represented as the mean of 4 independent experiments +/− SEM. *p-value <0.05, **p-value <0.01 and ***p-value <0.001 with a Mann-Whitney test followed by Bonferroni correction for comparisons of the control vs the treatments. For analysis of apoptosis, cells were stained using a TUNEL assay, and the percentage of apoptotic cells was assessed with an Array Scan VTI. The results are expressed as the percentage of TUNEL-positive cells compared to total c [file 12964_2017_182_MOESM1_ESM.tif]

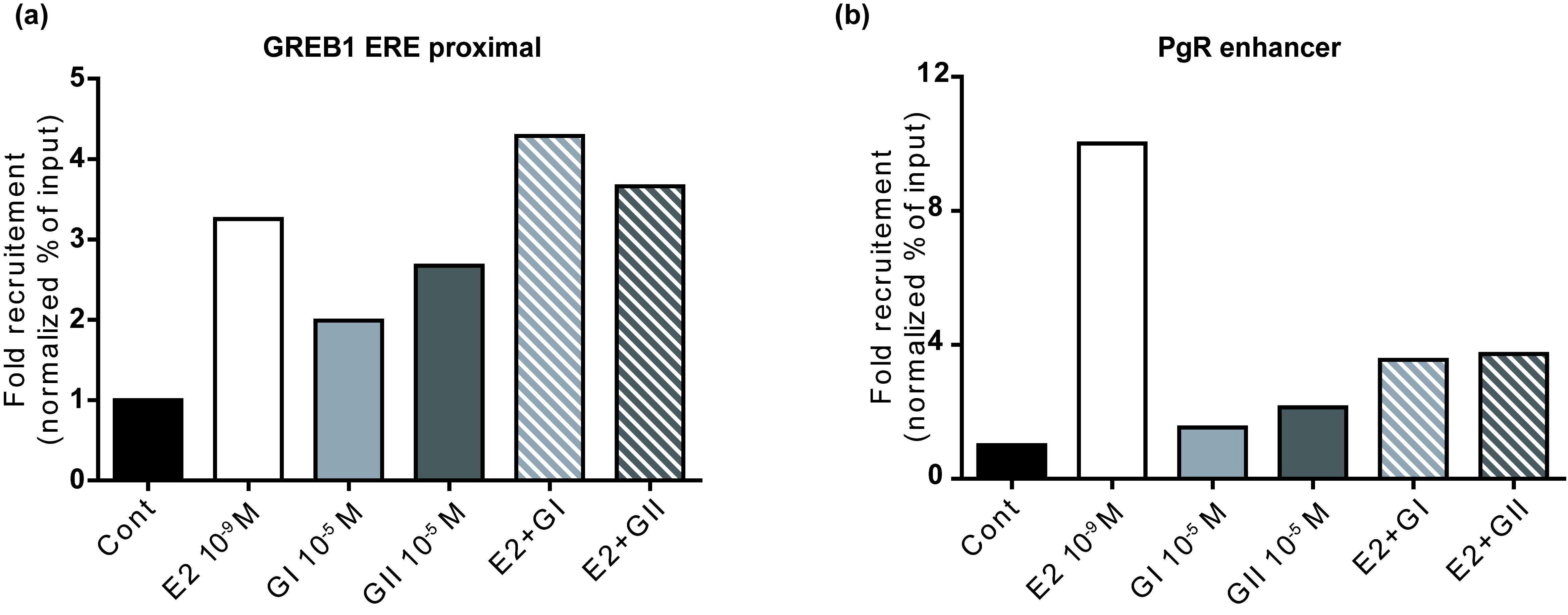

Supplement: Supplementary file 3 — Effect of glyceollins on ERα recruitment on GREB1 promoter and PgR enhancer. MCF-7 cells were treated with vehicle (black), 10−9 M E2 (white), 10−5 M glyceollin I (light grey) and II (hard grey), or a combination of E2 and each of the glyceollins (hatched squares). The recruitment of ERα on GREB1 promoter (a) and PgR enhancer (b) was assessed by chromatin immunoprecipitation followed by real time PCR. Results are expressed in fold recruitment compared to control and are the mean of two independent experiments. (TIFF 940 kb) [file 12964_2017_182_MOESM3_ESM.tif]

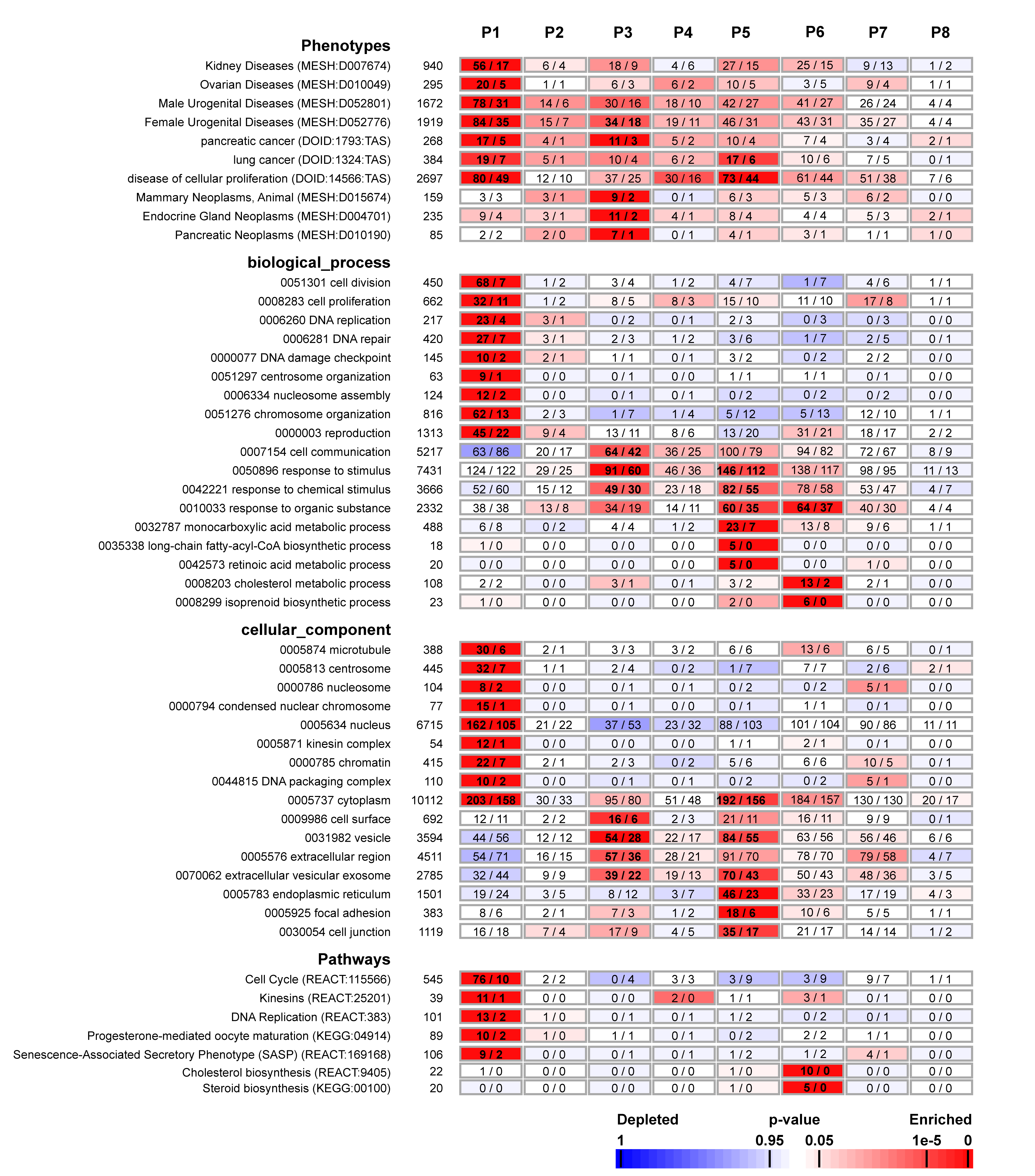

Supplement: Supplementary file 5 — GO enrichment analysis of different treatment-related expression patterns. Eight expression patterns are matched with a selection of GO terms from the ontology “phenotypes,” “biological process,” “cellular component” and “pathways.” The numbers of genes associated with each GO term are indicated in the first column. Enrichment is indicated by bolded rectangles, where the first number indicates the number of genes found in our analysis and the second the number expected with a random list of genes. Overrepresented genes in a specific GO term are shown in red, and underrepresented genes are shown in blue. (TIFF 2724 kb) [file 12964_2017_182_MOESM5_ESM.tif]

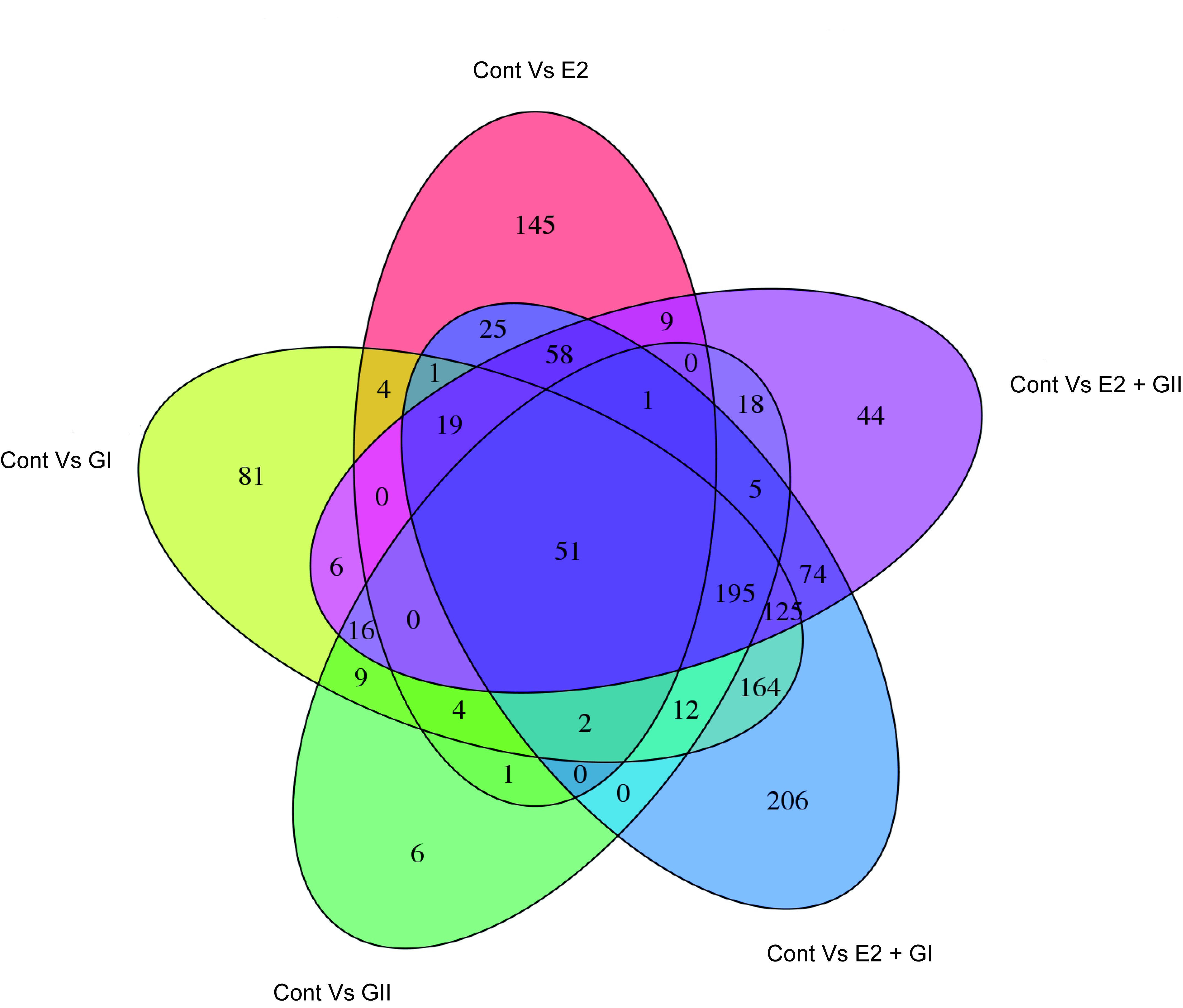

Supplement: Supplementary file 6 — Venn diagram. A Venn diagram was created from the list of differentially expressed genes obtained from comparisons of the control and E2 (red), GI (yellow), GII (green), E2 + GI (blue) and E2 + GII (purple) treatments. (TIFF 3761 kb) [file 12964_2017_182_MOESM6_ESM.tif]
