# Supplementary material for: Glyceollins trigger anti-proliferative effects through estradiol-dependent and independent pathways in breast cancer cells
Source: Cell Commun Signal. 2017 Jun 30;15:26. doi: 10.1186/s12964-017-0182-1 (PMC5493871; doi:10.1186/s12964-017-0182-1)
Supplement: Supplementary file 2 — Supplemental material. (PDF 157 kb) [file 12964_2017_182_MOESM2_ESM.pdf]

Supplemental material

Cell cycle analysis

MCF-7 cells (100,000 cells/well) were plated in 6-well plates. After 72 h of serum and steroid deprivation, the cells were treated for 72 h with solvent as control, 10^{-9} M E2, 10^{-5} M glyceollin I or II, or a combination of E2 and glyceollin I or II. Then, the cells were trypsinized and fixed in 70% ethanol before staining with propidium iodide. The percentage of cells in each cell cycle phase was assessed by flow cytometry with a FACS Calibur (BD Biosciences).

Measurement of apoptosis

MCF-7 cells (4,000 cells/well) were plated in 96-well plates. After 72h of serum and steroid deprivation, the cells were treated for 72 h with solvent as control, 10^{-9} M E2, 10^{-5} M glyceollin I or II, or a combination of E2 and glyceollin I or II. TUNEL staining was assessed with an In Situ Cell Death Detection Kit, Fluorescein (Roche) according to the manufacturer's instructions. The fluorescence and percentage of TUNEL-positive cells were determined with an Array Scan VTI (Thermo Fisher Scientific) on the ImPACcell platform (Rennes, France).

Chromatin Immunoprecipitation (ChIP)

MCF-7 cells (2,000,000 cells /dishes) were plated in 10cm dishes and then deprived of steroids and serum for 72 h. The cells were treated for 1h with 10^{-9} M E2, with 10^{-5} M GI or GII with or without 10^{-9} M E2. Then, cells were cross-linked for 10 min with 1.5% of formaldehyde (Sigma). Cells were lysed in lysis buffer (50 mM Tris-HCl, pH 8.1, 10m M EDTA, 0.5% Empigen BB and 1% SDS). Chromatin was sonicated 10 min (15 sec on/off cycles) on Bioruptor (Diagenode) at highest intensity. Soluble chromatin was diluted in IP buffer (20 mM Tris-HCl, pH 8.1, 2 mM EDTA, 0.1% Triton X-100) with 2 µg of ERα antibody (E115, Abcam) and yeast RNA as non-specific competitor and incubated overnight at 4°C on rocking platform. Then, protein G coupled sepharose beads were added to the samples and were incubated 4h à 4°C. Immune complexes were washed one time in washing buffer 1 (20 mM Tris-HCl, pH 8.1, 2 mM EDTA, 150 mM NaCl, 1% Triton X-100 and 0.1% SDS), one time in washing buffer 2 (20 mM Tris-HCl, pH 8.1, 2 mM EDTA, 500 mM NaCl, 1% Triton X-100 and 0.1% SDS), one time in washing buffer 3 (10 mM Tris-HCl, pH 8.1, 1 mM EDTA, 250 mM LiCl, 1% Deoxycholate and 1% NP-40) and finally two times in washing buffer 4 (10 mM Tris-HCl, pH 8.1, 1 mM EDTA). After washing, immune complexes were extracted with 100µl of extraction buffer (0.1 M $NaHCO_3$ and 1% SDS). Cross-linking was reverse by incubation of samples overnight at 65°C and DNA was purified using the Nucleospin Gel and PCR cleanup kit (Macherey Nagel).

Enrichment analysis on the ERE proximal of GREB1 (Fwd: CACTTTGAGCAAAAGCCACA and Rev: GACCCAGTTGCCACACTTTT) and on an enhancer 1 of PgR described in [58] was normalized using an irrelevant region on the chromosome 10 (Fwd: AGGTGACAAGCCAAGTGTCC and Rev: GCCTGGTGGCATACTAAAGG). Analysis was performed by real time PCR on a CFX 384 apparatus (BioRad) on 2µL of immunoprecipitation or 0.2µL of input with 500nM of primers and iTaq Universal SYBR Green Supermix (BioRad).
